# Evaluation of facilitative supervision visits in primary health care service delivery in Northern Ghana

**DOI:** 10.1186/1472-6963-13-358

**Published:** 2013-09-24

**Authors:** Moses Aikins, Amos Laar, Justice Nonvignon, Samuel Sackey, Takaharu Ikeda, George Woode, Alexis Nang-Beifubah, Frank Nyonator

**Affiliations:** 1 School of Public Health, College of Health Sciences, University of Ghana, Legon, Accra, Ghana; 2 Japanese International Cooperation Agency, Ghana Office, Accra, Ghana; 3 Regional Health Directorate, Ghana Health Service, Upper West Region, Accra, Wa, Ghana; 4 Policy Planning, Monitoring and Evaluation Division, Ghana Health Service, Accra, Ghana

**Keywords:** Facilitative supervision visit, Primary health care, Service delivery, Ghana

## Abstract

**Background:**

In Ghana’s health delivery services, facilitative supervisory visit (FSV) as a system of management is new. This paper presents the standard evaluation results of FSV, which formed an integral part of the community-based health planning services (CHPS) initiative.

**Methods:**

The study was conducted in the Upper West Region of Ghana. The Project developed guidelines and tools for FSV for four different health system levels – regional, district, sub-district and community levels. Electronic data from all four levels representing quarterly results were compiled into their annual equivalents, and summarized graphically for comparison.

**Results:**

The data show that all the nine districts embraced the FSV concept even though they differed markedly with regard to the degree of adherence to some set benchmarks. Three DHMTs (Wa Municipal, Lawra and Jirapa) were graded as good while the remaining six DHMTs were adjudged as fair in relation to management of supplies, transport and equipment, information, meeting, and technical support.

**Conclusions:**

The data further suggest that there is much to gain both individually and institutionally from FSVs. Generally, FSVs are crucial to the delivery of primary health care services in especially rural areas.

## Background

Primary Health Care (PHC) in Ghana is structured to serve the rural and urban population according to priority [1]. The evolution of the PHC programme in Ghana has been well documented [2-4] from the Danfa Comprehensive Rural Health and Family Planning project (1970 – 1977), Primary Health Care Strategy paper of 1977/78, WHO-sponsored Brong-Ahafo Regional Development project to the Bamako Initiative.

Currently, PHC services are provided through the Community-based Health Planning Services (CHPS), which emerged out of the experimental study of the Navrongo Health Research Centre (NHRC) [5]. The CHPS is a priority programme especially for rural areas, which are mostly deprived of permanent health infrastructures [1]. The Government of Ghana adopted the CHPS programme in 1999 as a strategy to redress the inequality in access to health services by strengthening community health services. Notwithstanding the commendable efforts of this programme, access to PHC services is still limited, especially in rural areas, where the expansion of CHPS has been painfully slow. The many reasons for this slow pace of events include lack of political will for scale up, inadequate resources, lack of administrative capacities, inadequate number, as well as competent Community Health Officers (CHOs), suboptimal levels of local people’s participation and different understanding of the concept of CHPS amongst health sector leadership [6].

Through its bilateral cooperation, the Government of Ghana requested technical support from the Government of Japan for the expansion of CHPS. In response to this, JICA implemented a project to support the expansion of CHPS for the purpose of strengthening community health services called "The Project for the Scaling-up of CHPS Implementation in the Upper West Region (UWR)". The target area of the Project was the UWR, the region of extreme poverty where access to PHC services is inadequate and health indicators, especially infant mortality rate, are worse than the other parts of Ghana. The Project started in cooperation with Japan Overseas Cooperation Volunteers (JOCV), the Japanese Grant Aid project, and an expert at the Ministry of Health, Ghana. Facilitative supervision visits (FSV) form an integral and crucial part of this project as the success of the CHPS approach to PHC delivery largely depends on supervision.

Facilitative supervision is a system of management whereby supervisors at all levels in an institution focus on the needs of the staff they oversee. Supervisors who use the facilitative approach consider staff as their customers. The most important part of the facilitative supervisor’s role is to enable staff to manage the quality improvement process, to meet the needs of their clients, and to implement institutional goals. This approach emphasizes monitoring, joint problem solving, and two-way communication between the supervisor and those being supervised [6,7]. Adoption of a facilitative approach leads to a shift from inspection and fault-finding to assessment and collective problem solving to continuously improve the quality of care. Studies have shown that (supportive) supervision of PHC workers could potentially improve health system outputs - through motivation and job satisfaction [6,8] and ensure that quality assurance processes are sustainable [9].

This paper presents the standard evaluation results of FSV in terms of its format, way of implementation, as well as its status in all the nine districts of the UWR. The status of FSV covers scores for report to the Regional Health Management Team (RHMT), management of supplies, management of transport and equipment, information management, management of meeting, technical support to sub-District Health Management Team (SDHT) and overall scores for the implementation period.

## Methods

### Study area

The UWR has a population of about 671,043 and a total area of 18,476 km^2^. The inhabitants are mainly traders and farmers. In 2010, the region has nine districts, each having a District Health Management Teams (DHMT). The nine districts are further demarcated into 65 sub-districts and 197 CHPS zones (as at 2009). Since implementation in 2007, there has been gradual growth in number of functional CHPS zones and as at 2010 there were 94 functional CHPS zones [10]. Feedback from these zones reaches the RHMT through a three-tier reporting system.

The RHMT comprises four main units (Public Health, Clinical Care, Health Administration and Support Services), and the Office of the Regional Director. The four units are responsible for strategic planning, resource mobilization and distribution, training, technical support, monitoring and evaluation of service delivery in the districts. There are 4 public hospitals, 4 Mission hospitals, 1 private hospital, 61 health centres, 4 clinics, 2 reproductive health centres, 4 private maternity homes and 4 private clinics in the region. There are also at least 885 traditional birth attendants (TBAs), 2,492 community-based surveillance volunteers (CBSVs) and184 guinea worm eradication campaign volunteers who are providing services in the communities with supervision from sub-district health staff [9]. Community participation in health delivery is facilitated at all levels through community representation on various health committees at regional, district and sub district levels.

### Intervention

The Project for Scaling up of CHPS Implementation in the Upper West Region was implemented in March 2006, for four years duration and ended in March 2010. The Project assisted the Ghana Health Service to scale up CHPS implementation in the region. The Project reviewed the conventional monitoring system within CHPS implementation. Based on the results and discussions with stakeholders, guidelines and tools for FSV were developed and trainings conducted for RHMT, DHMT, SDHT and CHO. After the trainings, FSV was introduced in all the 9 districts in the region. The FSV programme covered RHMT, DHMT, SDHT and CHO. The four identifiable processes of FSV at the different levels are described as follows.

#### RHMT self-monitoring

At the RHMT level, Self-Monitoring is done on quarterly basis. This involves a) Assessment by the Supervisory team; b) Submission of Supervisory Report (which should be immediate); c) Data analysis by the Health Information Officer; d) Report writing (analysis report) by the Supervisory team; and e) Reporting to Regional Director of Health Services (RDHS) on monthly basis during the RHMT meeting.

#### FSV of DHMT by RHMT

This occurs at the DHMT level and is conducted by the RHMT. It involves the following processes: a) Supervision by the RHMT Supervisory Team; b) Submission of Supervisory Report which should be immediate to the RDHS; c) Data analysis by the Health Information Unit; d) Report writing (Feedback report) by the Supervisory team in two forms – Analysis Report for each individual district and an integrated analysis report for all districts every 6 months; e) Reporting to RHMT/RDHS on monthly basis during the RHMT meetings; and f) Feedback to DHMTs, which comprises of monthly Feedback reports to each district and an integrated half-year report made through presentation by RHMT. Facilitative supervision over the DHMT is done quarterly by the RHMT Supervisory team. A supervisory report is immediately submitted to the RDHS. The Regional Health Information Officer analyzes the data collected and presents it in tabular form. The supervisory team then write analysis report for each District the following month and for all districts integrated report every six months. The team report to the RHMT/RDHS at the monthly RHMT meeting. A feedback is sent to each district the next month and to all districts during the half-year and annual review meeting through presentations by the RHMT.

#### FSV of SDHT by DHMT

This is conducted by the DHMT on its SDHT. It involves the following processes: a) Supervision by the DHMT Supervisory Team; b) Submission of Monitoring Sheets and Supervisory Report which should be immediate to the District Director of Health Service (DDHS); c) Data analysis (tabulation form) by the Health Information Unit; d) Report writing (Analysis report) by the Supervisory team; e) Feedback to the SDHT in two forms – Monthly Analysis Report for each individual CHPS zone and an integrated analysis report for all CHPS zones to be presented at monthly CHPS Review meetings and also at Quarterly Review meetings through presentation; and f) Submission of copies of Analysis Report to RHMT on the 15^th^ day of every month through the DHMT. Facilitative Supervision over SDHT was conducted quarterly by the DHMT Supervisory Team. After FSV by the DHMT, the monitoring sheet and supervisory report were immediately submitted to the DDHS. Data collected was analysed by the District’s Health Information Officer and presents it in a tabular format. The supervisory team then sends the feedback analysis report to each SDHT the following month of FSV and to all SDHT (integrated) during monthly CHO review meeting or quarterly review meeting through presentations. The DHMT concurrently presents to the RHMT copies of the analysis report on the 15th of every month.

#### FSV of CHO by SDHT

This final stage is conducted by the SDHT on the CHOs. It involves the following processes: a) Supervision of CHOs by the SDHT Staff member; b) Submission of Monitoring Sheets and Supervisory Report to the DHMT on the 5^th^ of every month by the SDHT Staff member who undertook the supervision; c) Data analysis (tabulation form) by the Health Information Unit at the DHMT; d) Report writing (Analysis report) by the Supervisory team at the DHMT; e) Feedback to the SDHT in three forms namely, i) Immediate Feedback by SDHT to each CHO; ii) Submission of monthly Analysis Report on each CHO; and iii) an integrated analysis report on all CHOs to be presented at monthly CHPS Review meeting and also at Quarterly Review meeting through presentation by the DHMT Supervisory Team; and f) Submission of copies of Analysis Report to RHMT on the 15^th^ day of every month through the DHMT. Facilitative Supervision of Community Health Officers (CHOs) is done monthly by SDHTs staff members. Copies of the monitoring sheet and supervisory report are submitted to the respective DHMTs on the fifth (5th) of every month by the SDHT staff members. Data collected are analysed by the District’s Health Information Officer. The supervisory team at DHMT then write analysis report. Through this analysis report, an immediate feedback is given to each CHO during the FSV of the following month. Furthermore, feedback is given to all CHO during monthly CHPS review meeting and quarterly review meetings. The DHMT subsequently report to the RHMT on the 15th of every month.

**Figure 1:**
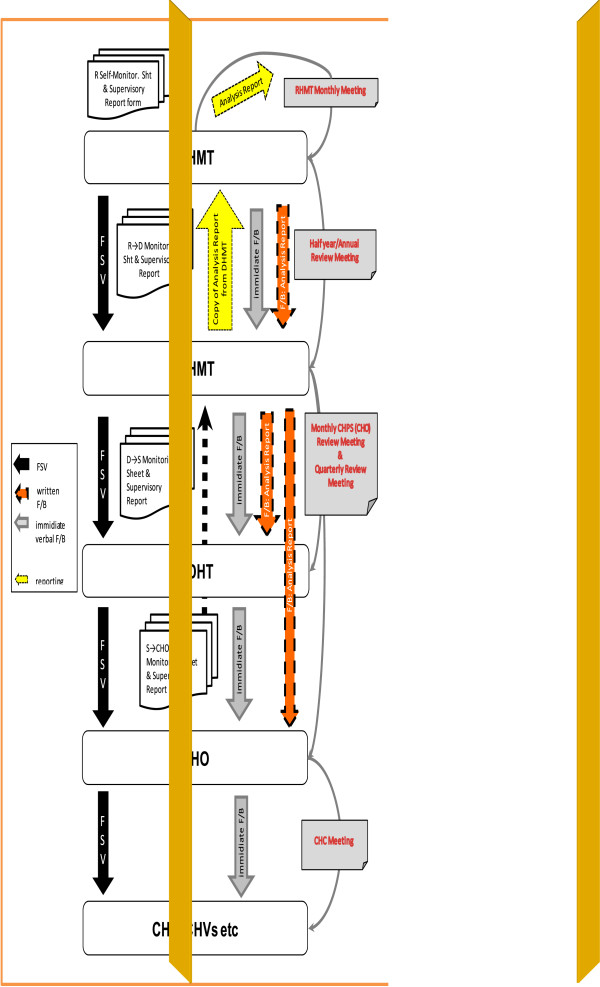
Diagrammatic representation of facilitative supervision visit at all levels.

Figure 1 shows the diagrammatic representation of FSV among the various component of the programme indicating responsibilities, reporting routes, verbal and written feedbacks and review meetings. This also shows the monthly or quarterly responsibilities of each health administration team. The District Information Officers of all the 9 districts produce various reports depending on the circumstance: monthly feedback reports for DHMTs and SHDTs, quarterly and annual reports for RHMT, DHMTs and SDHTs, and presentations at review meetings.

### Data collection

The evaluation data was collected between July 2010 and August 2010 from all the 9 districts of the UWR. The data collection techniques used was electronic data extraction during field visits.

#### FSV electronic data

With the assistance of Regional Information Officer and the District Information Officers, FSV electronic data was collected on the RHMT self-monitoring, FSV data by RHMT over DHMT, FSV data by DHMT over SDHT, and FSV data by SDHT over CHOs from April 2009 to March 2010 were collected. The FSV data capture is built in Microsoft Excel software.

#### Field visits

Twenty-seven (27) CHPS zones randomly selected with the assistance of the DHMTs were visited. Three (3) CHPS zones were visited in each district. At the CHPS zones, data was verified from their records.

### Data analysis

Data collection, cleaning, updating and compilation were done between 5^th^ July and 31^st^ July 2010 in the Upper West Region. The evaluation was conducted in August 2010. The FSV assessment was based on 'yes’ and 'no’ responses to a series of questions relating to the identified issues for the DHMT. The issues were reports to RHMT, management of supplies, management of transportation and equipment, information management, management of meetings and technical support to SDHT. The identified issues for the SDHT and CHOs are listed in Tables 1 and 2. These questions explore whether a scheduled activity or event under these issues took place as planned within the period (usually in the last three months). Each question is scored according to the answer given. The scores are quantified using the developed scales in the area. The final score is standardised and value returned as a percentage. The final scores are then graded into ordinal scale of 'good’ , 'fair’ and 'poor’ with their equivalents in percentage scale as in Tables 1 and 2. The assessment was done every quarter, and the completed forms submitted to the District Information Officer for computerization. The disaggregated quarterly results of all the districts were compiled and summarised into their annual equivalents.

**Table 1:** Areas of FSV assessment of SDHT by DHMT

**No.**	**Issues/areas**	**Assessment score**
1	Timely submission to RHMT	Four scale score: Excellent = 4; Good = 3; Fair = 2; and Poor = 1
2	Management of supplies	Three scale score: Good = 2; Fair = 1; and Poor = 0
3	Transport & equipment	Three scale score: Good = 2; Fair = 1; and Poor = 0
4	Information management	Three scale score: Good = 2; Fair = 1; and Poor = 0
5	Management meeting	Four scale score: Excellent = 9; Good = 8–7; Fair = 6–4; and Poor = 3
6	Technical support	Four scale score: Excellent = 10; Good = 9–8; Fair = 7–5; and Poor = 4
7	Referrals procedures	Four scale score: Excellent = 8; Good = 7–6; Fair = 5–4; and Poor = 3
	Final score	*Group 1:* Good: = ≥ 80%; Fair = ≥ 60%; and Poor: = ≤ 59%
		*Group 2:* Excellent: = ≥ 90%; Good: = 89 - 80%;
		Fair = ≥ 79 - 60%; and Poor: = ≤ 59%

**Table 2:** Areas of FSV assessment of CHO by SDHT

**No.**	**Issues/areas**	**Assessment score**
1	Condition of CHPS compound:	Three scale score: Good = 2; Fair = 1; and Poor = 0
	• Basic facilities	
	• Organization of rooms & documents (observed)	
2	Reporting & Documentation:	Three scale score: Good = 2; Fair = 1; and Poor = 0
	• Situation of reporting to SDHT	
	• Statistical report	
	• Logistic related documents (check tally cards, ledger book, requisition, motorcycle log sheet/book)	
3	Program based activities:	Three scale score: Good = 2; Fair = 1; and Poor = 0
	• Health promotion	
	• Home visits	
	• Reproductive health (reproductive health, school health, vitamin A, adolescent health services)	
	• Immunization	
	• Child Welfare Clinic	
	• Morbidity (malaria, ARI, diarrhoea, others)	
4	Family planning	Three scale score: Good = 2; Fair = 1; and Poor = 0
5	Community based surveillance	Three scale score: Good = 2; Fair = 1; and Poor = 0
6	HIV/AIDS consultation	Three scale score: Good = 2; Fair = 1; and Poor = 0
7	Other activities:	Three scale score: Good = 2; Fair = 1; and Poor = 0
	• Action plan & calendar of activities	
	• Community registers	
	• Supervise & support community health workers (activities done by CHO for communities, activities done by community with CHO’s attendance, supportive activities by community, Community Health Action Plan (CHAP))	
	• Referrals	
8	Equipment & supplies	Three scale score: Good = 2; Fair = 1; and Poor = 0
	• Essential equipment & its management	
	• Medical supplies & consumable goods	
	• Forms, books & register	
	Final score	*Group 1:* Good: = ≥ 80%; Fair = ≥ 60%; and Poor: = ≤ 59%
		*Group 2:* Excellent: = ≥ 90%; Good: = 89 - 80%;
		Fair = ≥ 79 - 60%; and Poor: = ≤ 59%

### Ethical issues

The study was undertaken with approval by the Policy Planning, Monitoring and Evaluation Division of the Ghana Health Service, the Upper West Regional Directorate and the District Health Directorates of all districts and sub-districts that took part in the study. The Ghana Office of the Japanese International Cooperation Agency also approved the study.

## Results

**Figure 2:**
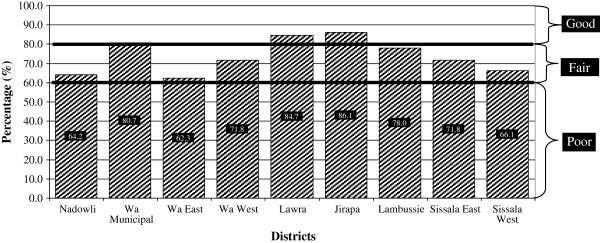
Overall FSV scores of DHMTs.

**Figure 3:**
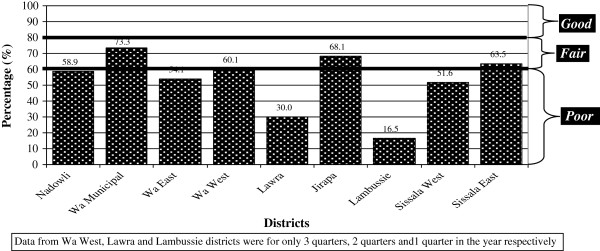
Overall FSV scores of SDHTs.

The total overall scores for the DHMT, SHDT and CHO issues are shown graphically (Figure 2 through Figure 3) for comparison.

### Facilitative supervision visits of DHMT

Table 3 shows the performance of each district during the year and their overall scores are presented in Figure 2.

**Table 3:** Annual district health management teams FSV scores

**Districts**	**Percentage (%) scores of:**						
	**Total overall**	**Report to RHMT**	**Management of supplies**	**Management of transportation & equipment**	**Information management**	**Management of meeting**	**Tech support to SDHT**
Nadowli	64.2	68.8	56.3	80.0	75.0	52.8	52.5
Wa Municipal	80.7	75.0	81.3	85.0	91.7	63.9	87.5
Wa East	62.3	56.3	62.5	72.5	64.6	55.6	62.5
Wa West	71.8	68.8	62.5	72.5	89.6	72.2	65.0
Lawra	84.7	87.5	84.4	85.0	85.4	86.1	80.0
Jirapa	86.1	75.0	93.8	95.0	97.9	75.0	80.0
Lambussie	78.0	100.0	78.1	82.5	77.1	61.1	70.0
Sissala East	71.8	87.5	81.3	80.0	58.3	63.9	60.0
Sissala West	66.1	62.5	53.1	77.5	81.3	50.0	72.5

The nine districts differ markedly with respect to their performance on the various items assessed. Jirapa DHMT scored the highest marks in management of supplies (93.8%), management of transportation and equipment (95%) and information management (97.9%). Lambussie scored the highest mark in report to RHMT (100%), Lawra scored highest mark in management of meetings (86.1%) and Wa Municipal scored highest in Technical support to SDMT (87.5%). Sissala West DHMT scored the lowest mark in three areas: report to RHMT (62.5%), management of supplies (53.1%) and management of meetings (50%). Using the overall scores in Figure 2, three DHMTs (i.e., 43% of DHMTs) were graded as good – Wa Municipal (80.7%), Lawra (84.7%) and Jirapa (86.1%). All the remaining six DHMTs were adjudged as fair (Figure 2).

### Facilitative supervision visits of SHMT

**Figure 4:**
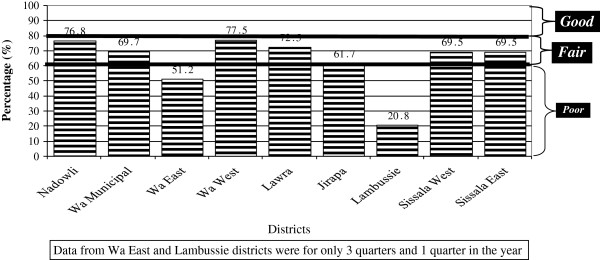
Overall FSV scores of CHOs.

The aggregated performances of the various SDHT for the study year are shown in Table 4. Wa Municipal SHMTs scored the highest marks in reports to DHMT (90.3%), management of supplies (87.5%), information management (88.5%) and technical support to CHOs (61.1%). Sissala East scored the highest mark in management of meetings (52.5%) and Sissala West scored highest mark in referrals (74.1%). Lambussie SDHTs scored the lowest mark in four areas: report to DHMT (23.2%), management of supplies (25%), information management (25%) and management of meetings (6.3%). Using the overall scores in Figure 4, none of the SDHTs were grade as good. Four of the nine districts SDHTs were, however graded fair – Wa Municipal (73.3%), Jirapa (68.1%), Sissala East (63.1%) and Wa West (60.1%). The remaining five district SDHTs were graded poor – Nadowli (58.9%), Wa East (54.1%), Sissala West (51.6%), Lawra (30%) and Lambussie (16.5%).

**Table 4:** Annual sub-district health teams FSV scores

**District**	**Percentage (%) scores of:**						
	**Total points**	**Reports**	**Management of supplies**	**Information management**	**Meetings**	**Technical support**	**Referrals**
Nadowli	58.9	77.4	71.7	69.7	33.6	42.5	58.6
Wa Municipal	73.3	90.3	87.5	88.5	51.0	61.1	61.6
Wa East	54.1	74.5	78.9	74.8	39.2	0.0	57.2
Wa West	60.1	64.1	66.8	74.0	43.1	0.0	52.6
Lawra	30.0	46.6	30.6	28.8	26.0	12.9	35.2

### Facilitative supervision visits of CHO

Table 5 shows that Wa West CHOs scored the highest marks in conditions of CHO compounds (84.1%), activities (76%), and equipment status (52.5%). Nadowli scored highest mark in documentation (95.3%) and medical supplies (80%) and Sissala East and West scored the highest mark in keeping of booklet (88.3% each). Lambussie CHOs scored the lowest mark in all the six areas assessed with marks ranging 0% to 25%. Using the overall scores in Figure 3, none of the CHOs were grade as good. Seven of the nine districts CHOs were graded as fair – Wa West (77.5%), Nadowli (76.8%), Lawra (72.5%), Wa Municipal (69.7%), Sissala West (69.5%), Sissala East (69.5%) and Jirapa (61.7%). The remaining two district CHOs were adjudged as poor – Wa East (51.2%) and Lambussie (20.8%).

**Table 5:** Annual CHO FSV scores

**Districts**	**Percentage (%) scores of:**						
	**Total points**	**Condition**	**Documentation**	**Booklet**	**Activities**	**Equipment status**	**Medical supplies**
Nadowli	76.9	81.4	95.4	87.7	69.12	47.56	80.0
Wa Municipal	69.7	65.3	91.0	87.2	60.8	36.8	77.0
Wa East	51.3	57.6	69.0	57.6	36.5	34.7	48.8
Wa West	77.5	84.1	92.5	85.5	76.0	52.5	74.7
Lawra	72.5	77.5	90.9	87.2	67.4	40.1	71.7
Jirapa	61.7	79.6	82.0	69.7	49.2	34.3	55.5
Lambussie	20.8	25.0	24.4	0.0	19.7	18.4	16.3
Sissala West	69.5	73.3	81.7	88.3	66.3	36.5	71.0
Sissala East	69.5	73.3	81.7	88.3	67.6	36.5	71.0

## Discussion

In the past couple of decades, theories have been advanced to challenge the traditional concept that assuring and improving quality is the result of external review, inspection of the end result, and a heavy investment in supervisors whose major function is to monitor staff [11]. In the recently proposed theories, the focus has shifted to anticipating and preventing problems rather than correcting them. Motivated by such theories, facilitative supervision, instead of finding fault and leveling blame at individuals, emphasized determining whether or not existing work processes are planned, designed, and implemented in such a way as to achieve the desired end result – providing a high-quality service that meets clients' needs. During the mid 1990s, Chambers and Long [12] in their paper that explored the theoretical and practical issues pertinent to the implementation and development of supportive clinical supervision showed that a facilitative approach to clinical supervision is therapeutic and self-propelling for both supervisor and supervisee [12].

In Ghana’s health delivery services, FSV as a system of management whereby supervisors at all levels in an institution focus on the needs of the staff they oversee is new. This paper presents the standard evaluation results of FSV, which formed an integral and crucial part of a Government of Ghana-Japan bilateral project implemented in the UWR of Ghana. The aim of the project was to support the expansion of CHPS to strengthen community health services in the region. Even though the CHPS as a priority programme designed for rural areas, which are mostly deprived of permanent health infrastructures [1], has chalked some successes, there have been some bottlenecks in terms of its scalability in the urban areas. If the adoption of FSV could improve CHPS’ efficiency and effectiveness irrespective of setting, it will contribute significantly to addressing some of our health care service delivery conundrums.

We show that all the nine districts enthusiastically embraced the FSV approach even though they differed markedly with regard to the degree of adherence to some set benchmarks. The benchmarks being referred to were developed taking into consideration management of supplies, management of transport and equipment, information management, management of meeting, technical support, and scores for report to the RHMT, DHMT, and SDHT. Previous studies have shown that CHPS is consistently associated with an increase of health care service delivery and receipt [1,4]. However, those studies did not address process-related indicators being referred to in this study. The results of the current study, offer a novel insight regarding the feasibility of incorporating FSV into the CHPS initiative. In Donabedian’s framework for quality of care assessment, three different components of quality of care are noted: structure, process, and outcome [13]. 'Structure’ refers to the attributes of the settings where health care occurs; 'process’ represents what is actually done in giving and receiving care (this is further divided in to interpersonal, and technical components); and 'outcome’ shows the effects of care on the health status of patients and populations. A successful implementation of FSV could have a positive impact on the 'process’ component of Donnabedian’s three-legged framework.

The structure of the health system in Ghana is such that DHMTs and SDHTs bear the responsibility of ensuring that primary health care services function. Therefore, the success of the CHPS strategy will, to some extent, depend on the performance of DHMTs and SDHTs in terms of facilitative supervision. The results of the study show that three DHMTs (Wa Municipal, Lawra and Jirapa) were graded as good while the remaining six DHMTs were adjudged as fair (Figure 2). Each of the areas of evaluation is crucial to the success of the CHPS strategy in the region. Therefore, improvements in each area would consequently improve service delivery through the CHPS approach. Typically, each DHMT has to implement strategies to work on their weaknesses while also improving (or at least maintaining its strengths).

Facilitative supervision visits were also conducted quarterly by the DHMT Supervisory Team over the SDHTs. The aggregated performances of the various SDHT for the study year show that Wa Municipal SHMTs scored the highest marks in reports to DHMT, management of supplies, information management and technical support to CHOs. Sissala East scored the highest mark in management of meetings and Sissala West scored highest mark in referrals. Lambussie SDHTs scored the lowest mark in four areas: report to DHMT, management of supplies, information management and management of meetings. Using the overall scores as shown in Figure 3, none of the SDHTs were grade as good and SDHTs in four districts performed poorly. The worst performance of SDHTs in districts relate to provision of technical support to CHOs; apart from SDHTs in Wa Municipal which scored 61% under technical support, SDHTs in all other districts scored below 50%. This poor performance under technical support calls for concern, since technical support is a key area that directly affects the activities of the CHOs (apart from supplies), who require support regularly to enable them function well in the communities.

The base of the primary health care system is the community level, where CHOs operate the CHPS compounds. A recent study in the Upper East Region of Ghana shows that supervision (especially one in which supervisor provides some support to the one being supervised) improves the productivity of health care workers at the CHPS level [8]. For the productivity of CHOs in the Upper East Region to improve, SDHTs need to step up their performance. This is a key factor in raising health system output. Another observation is that the performance of DHMTs in terms of facilitative supervision visits does not seem to reflect in the performance of the SDHTs under the districts. The challenge is for the various DHMTs to step up their monitoring to ensure that SDHTs improve upon their performances.

Facilitative Supervision of CHOs is done monthly by SDHTs staff members. Overall, CHOs in all districts scored above 50%, with the exception of CHOs in Lambusie district who scored below 20%. The observed low performance of CHOs and SDHT in Lambusie in relation to FSV requires further investigation.

This pilot program in the UWR suggests that there is much to gain both individually and institutionally, from a transition to more facilitative styles of supervision. It is, however, almost axiomatic that institutions and organizations particularly in the field of health care where medical hierarchies dictate a conventional supervisory approach, may find - a shift from the conventional approach to this new approach daunting. Some may believe that the conventional approach of policing employees has stood the test of time and does not need alteration. Others may believe that changing to the FSV approach will take more time, resources, thought, and attention than is possible, given the level of resources available. Yet, others fear that management will lose their directive role when supervisory systems change from inspection to facilitative. We provide evidence to the contrary. In the UWR, management at the various districts and at the regional levels remained responsible for planning and implementation of work, making available the facilities, training, and other resources needed to achieve their targets. To those who argue that the facilitative approach will be resource-intensive, some activities in East Africa have shown that the approach is possible even in very resource-poor settings [14].

Other institutions elsewhere have evidence of the feasibility of the approach. In a number of countries notably in Bangladesh, Kenya, Tanzania, Zimbabwe, and Uganda, the fruits of FSVs are evident. In the 1990s, Tanzania witnessed a rapid expansion of a family planning program as a result of the approach. Facilitative supervisors from the Tanzanian Ministry of Health serving as middle managers and trainers worked to introduce and implement protocols for clinical methods and assisted in resource management. They also helped to leverage additional resources for family planning services [15]. They also showed that in a facilitative supervisory fashion, high level practicum instructors were usually able to offset the effects of low level individual supervisors. They demonstrated that only those counselling trainees who encountered individuals functioning at high levels of facilitative conditions were able to grow on these dimensions.

That said we find it essential to do some nuancing of our study outcomes. First, we acknowledge that it will be unreasonable to wholly attribute the successes herein presented to the facilitative supervisory visits. The leadership provided by other investments by JICA in the CHPS, inputs from other stakeholders such as traditional leadership, and the district assembly could have contributed to this success story. In fact, the introduction of CHPS into districts occurs through extensive planning and community dialogue on the part of the health service and the community. A key principle of CHPS’ introduction is that traditional leaders of the community must accept the CHPS concept and commit themselves to supporting it. CHPS relies on participation and mobilization of the traditional community structure for service delivery. In this regard, the review by the Evidence Review Team 1 of the US Government Evidence Summit [16] on the theme: "Which community support activities improve the performance of CHWs?" is a relevant resource.

### Study limitations

At this point, it is important to state two limitations that our study may suffer from. First, it was beyond the remit of our evaluation to measure whether or not the process-level desirable attributes of the facilitative approach translate into measureable service outcomes. Earlier studies provide evidence in line with this possibility. For instance, Kim et al. [17] showed that supportive supervision reinforces prompt reflection and learning, helping novice health personnel to improve their interpersonal communication skills (Kim et al., 2002). Even though this current study could not measure this relationship directly, we are confident that the findings of Kim et al. [17] will apply in our setting.

Second, we do acknowledge the inherent deficiencies with stakeholder response data. For instance, our data could have been biased by reliance on response data provided by participants in programme management, who might lack objectivity and independence in assessing the topics under review. It is, however, reasonable that the results, which are based not on some, but on all the health districts of the region, and collected through a triangulation of two methods, have considerable relevance.

## Conclusions

Our study suggests that FSV is crucial to the delivery of primary health care services especially rural areas. However, evaluation of these visits using the criteria utilized in this study are even more important in ensuring that officers delivering PHC services and those supervising such delivery (at various levels) perform their duties. We particularly recommend that other regions implementing the CHPS approach take steps to implement FSV and an efficient way of evaluating such visits.

Even though facilitative supervision visits are important in the implementation of the CHPS strategy, it is also important to establish the relationship between these visits and final health outcomes. Further studies could explore this relationship. While acknowledging that the introduction of FSV will require change, and change may require time, we nevertheless believe that FSV is one approach, which may contribute significantly to improved and sustainable quality health services not only in the UWR, but in all parts of Ghana.

## Abbreviations

CBSV: Community based surveillance volunteer; CHO: Community health officer; CHPS: Community-based health planning and services; DDHS: District director of health services; DHMT: District health management team; FSV: Facilitative supervisory visit; JICA: Japanese international cooperation agency; JOCV: Japanese overseas cooperation volunteer; NHRC: Navrongo health research centre; PHC: Primary health care; RDHS: Regional director of health services; RHMT: Regional health management team; SDHT: Sub district health team; TBA: Traditional birth attendant; UWR: Upper west region.

## Competing interest

The authors declare that they have no competing interest.

## Authors’ contributions

MA, TI, GW conceived the study. MA and AL participated in the data collection. MA and SS undertook the data analysis. MA, AL and JN drafted the manuscript. MA, AL, JN, AN and FN revised the manuscript. All authors reviewed the final manuscript and gave approval.

## Authors’ information

MA is Vice Dean at the School of Public Health, University of Ghana and the project team leader. AL, JN and SS are Lectures at the School of Public Health, University of Ghana and members of the project team. TI and GW are staff of Japanese International Cooperation Agency, Ghana Office and supervised the project. AN is Upper West Regional Director of Health services and supervised the field work. FN was Director, Policy Planning, Monitoring and Evaluation Division, Ghana Health Service and involved in the initial implementation of the CHPS strategy.

## Pre-publication history

The pre-publication history for this paper can be accessed here:

http://www.biomedcentral.com/1472-6963/13/358/prepub
